# Biomarkers in Bladder Cancer: Present Status and Perspectives

**Published:** 2007-03-27

**Authors:** Wun-Jae Kim, Soongang Park, Yong-June Kim

**Affiliations:** 1 Department of Urology, Chungbuk National University, College of Medicine and Institute for Tumor Research, South Korea; 2 Department of Urology, Chungbuk National University, College of Medicine, Cheongju, South Korea

**Keywords:** Bladder cancer, Biomarker, Prognosis

## Abstract

Bladder cancers are a mixture of heterogeneous cell populations, and numerous factors are likely to be involved in dictating their recurrence, progression and the patient’s survival. For any candidate prognostic marker to have considerable clinical relevance, it must add some predictive capacity beyond that offered by conventional clinical and pathologic parameters. Here, the current situation in bladder cancer research with respect to identification of suitable prognostic markers is reviewed. A number of individual molecular markers that might predict bladder cancer recurrence and progression have been identified but many are not sufficiently sensitive or specific for the whole spectrum of bladder cancer diseases seen in routine clinical practice. These limitations have led to interest in other molecular parameters that could enable more accurate prognosis for bladder cancer patients. Of particular interest is the epigenetic silencing of tumor suppressor genes. Since the methylation of these genes can correlate with a poor prognosis, the methylation profile may represent a new bio-marker that indicates the risk of transitional cell carcinoma development. In addition, bladder cancer research is likely to be revolutionized by high-throughput molecular technologies, which allow rapid and global gene expression analysis of thousands of tumor samples. Initial studies employing these technologies have considerably expanded our ability to classify bladder cancers with respect to their survivability. Future microarray analyses are likely to reveal particular gene expression signatures that predict the likelihood of bladder cancer progression and recurrence, as well as patient’s survival and responsiveness to different anti-cancer therapies, with great specificity and sensitivity.

## Introduction

Currently, patients with bladder cancer are monitored for cancer recurrence or progression by periodic cystoscopy, the frequency of which varies depending on the risk factors associated with the disease. The assumption is that frequent cystoscopies facilitate the treatment of recurrences at an early stage, thereby potentially slowing the progression of the disease to muscle invasive disease. It has also been shown that assessment of the urine for the presence of particular cells or various bladder tumor markers can help prediction of tumor recurrence, progression, and metastasis. Therefore, inspection of urine samples can forecast the patient’s survival and response to various treatments. Urine cytology is a highly specific, noninvasive adjunct to cystoscopy that is quite sensitive in detecting high grade bladder cancers. However, it has poor sensitivity in detecting low grade disease, and its accuracy is dependent on the pathologist’s experience.

To date, numerous potential markers from patient’s serum, bladder washes, urinary specimens, and cancer tissues have been identified by a variety of molecular biology and genetic studies. Molecular markers such as Ki-67 and p53 do appear to have some promising correlations with bladder cancer development, but their predictive value remains to be conclusively verified. This review focuses on recent advances in bladder cancer research with respect to the identification of suitable molecular prognostic markers.

## Chromosomal Markers

Cytogenetic studies have identified many changes in the structure and copy number of chromosomes in transitional cell carcinomas of the urinary bladder. For example, loss of heterozygosity (LOH) studies have shown that the loss of 17p, 3p, 13q, 18q, or 10q is found more frequently in high grade, high stage bladder cancer (Knowles, 2001). Moreover, loss of 9q was observed in low as well as high grade bladder cancer, which suggests that the loss of 9q may be a primary event in the genesis of bladder cancer (Tsai et al. 1990). In superficial bladder cancers, early changes include deletions of 11p and 8p, and gains of 8q and 1q (Fadl-Elmula et al. 2001). Such superficial noninvasive papillary lesions do not exhibit LOH of 17p, unlike invasive cancers, 60% of which exhibited LOH of 17p (Olumi et al. 1990; Presti et al. 1991). Thus, LOH of 17p may participate in the progression of bladder cancer. However, the prognostic value of these changes in chromosome copy numbers requires further large prospective studies of patients with bladder cancer. Indeed, it has been suggested that although copy number alterations have been associated with progression-free survival, they are not independent prognostic factors for disease progression (Pycha et al. 1999).

Many tumors also bear DNA replication errors (RERs), where bases in simple mononucleotide or dinucleotide repeat sequences (microsatellites) have been added or deleted, when compared to the matching DNA of normal tissue specimens (Thibodeau et al. 1993). These RERs, which result in so-called microsatellite instability (MSI), arise from dysfunction of the DNA mismatch repair genes (*hMSH2*, *hMLH1*, *hMSH6*, *PMS1*, and *PMS2*). These alterations sometimes occur in microsatellites residing in the coding sequence of important growth regulatory genes and thereby contribute to the development of cancer (Peltomaki and de la Chapelle, 1997). One study has shown that microsatellite alterations in bladder cancer are associated with invasive cancer (Sardi et al. 1999), but this finding requires further verification. Meanwhile, many other studies have examined whether the shifted DNA bands in exfoliated urine or bladder wash cells that indicate MSI may be useful in the early diagnosis and follow up of bladder cancer (Uchida et al. 1996; Larsson et al. 2001; Seripa et al. 2001). However, it should be noted that microsatellite alterations in urine are indicators not only of malignancy but also of inflammatory conditions (Christensen et al. 2000). Thus, the search for MSI can only complement current diagnostic methods.

## Genetic Markers

Many of the genetic markers that are associated with bladder cancer have been subjected to extensive studies examining their biological roles in bladder cancer development and progression. Here, we focus on the more promising prognostic markers. These include proto-oncogenes/oncogenes, tumor suppressor genes, cell cycle regulators, and cell adhesion molecules (Table 1).

### Proto-oncogenes/oncogenes

Several investigators have shown a positive correlation between overexpression of epidermal growth factor receptor (EGFR) [as detected by immunohistochemistry (IHC)] and high grade, high stage bladder cancer, which suggests that EGFR expression is an independent prognostic factor that indicates advanced bladder cancer (Neal et al. 1985; Messing, 1990; Nguyen et al. 1994; Lipponen and Eskelinen, 1994). The product of the *HER-2/neu* (*c-erb-B2*) oncogene was also found to be frequently overexpressed in urinary bladder cancer (Lipponen et al. 1991) and to correlate with increased tumor grade, lowered cancer-specific survival, and a higher incidence of metastatic disease (Sato et al. 1992). However, different studies examining the prognostic significance of HER-2/neu protein expression levels in bladder cancer came to varying conclusions:some suggested a better or worse prognosis, while others failed to detect any prognostic relevance (Gandour-Edwards et al. 2002).

Genetic studies have also shown that *FGFR3* point mutations occur frequently in bladder cancers (Cappellen et al. 1999; van Rhijn et al. 2001). Since these *FGFR3* mutations occur particularly often in low grade and low stage bladder tumors, they could have prognostic value (van Rhijn et al. 2003). Another proto-oncogene that is frequently overexpressed in high grade bladder cancers is *c-myc*. However, these changes in c-myc expression do not appear to correlate with recurrence, progression, or survival (Kotake et al. 1990; Lipponen, 1995; Schmitz-Drager et al. 1997). Therefore, c-myc overexpression in bladder cancer may be of little prognostic significance.

### Tumor suppressor genes

p53 plays a key role in regulating cell cycle progression and apoptosis under genotoxic conditions and *p53* gene mutations are the most commonly occurring genetic defect found in human cancers (Hollstein et al. 1991). In bladder cancer, *p53* gene mutations are generally believed to indicate invasive bladder cancer and disease progression and to be useful as chemotherapy response markers, although some studies have come to contradictory conclusions (Schmitz-Drager et al. 2000; Sarkis et al. 1995; Sengelov et al. 1997). Another interesting tumor suppressor gene is *Rb*. Cordon-Cardo et al. (1992) have reported that patients with Rb-normal bladder tumors have a higher overall survival, independent of stage, than patients with Rb-altered tumors. Logothetis et al. (1992) also reported that of their group of patients with locally advanced bladder cancer that had been treated by surgery and chemotherapy, the patients whose tumors expressed an altered Rb protein had significantly poorer tumor-free survival rates. These observations were supported by Cote et al. (1998), who examined the IHC expression of the p53 and Rb proteins from the bladder cancers of 185 patients who had undergone radical cystectomy. This study found that patients whose tumors had altered expression levels of both p53 and Rb had significantly increased recurrence rates and decreased survival compared with those showing normal expression levels. Patients with altered expression levels of only one of these proteins had intermediate rates of recurrence and survival. Although this was a retrospective study that examined only a relatively small number of subjects, the results nevertheless indicate that dual analysis of Rb and p53 protein may improve the prognostic significance of p53 expression.

### Cell cycle regulators

Among the patients whose bladder cancers showed altered p53 expression levels, some patients also exhibited negative p21 expression. Those patients that were p21 negative and abnormal for p53 expression had a significantly higher recurrence rate and worse survival compared with those whose tumors were p21-positive (Stein et al. 1998). However, the prognostic value of p21 expression remains to be validated. Another interesting cell cycle regulator is p27. A multivariate analysis of patients with invasive tumors has revealed that decreased p27 expression is associated with poor overall and post-relapse survival (Korkolopoulou et al. 2000). Moreover, this study found that low p27 expression in combination with high Ki-67 expression was particularly strongly associated with poor overall survival (Korkolopoulou et al. 2000). Numerous multivariate analyses have found that Ki-67 by itself is also an independent prognostic marker of superficial bladder cancer progression and recurrence. In normal situations, IHC-detected Ki-67 antigen accumulates in the nuclei of proliferating cells from the G1 phase to mitosis but is absent from the nuclei of quiescent or resting cells (Gerdes et al. 1984). The studies examining the prognostic significance of Ki-67 expression have all yielded similar observations, which suggests this promising marker should be tested in larger prospective studies with standardized positive criteria (especially with regard to the cutoff value of the labeling index). However, the prognostic value of Ki-67 for patients with locally advanced or metastatic bladder cancer remains unclear.

With regard to the cyclins, changed expression levels of cyclin D1 have been shown to serve as an independent predictor of tumor recurrence, although they are not associated with disease progression (Wagner et al. 1999; Liukkonen et al. 2000). A study of a tissue microarray composed of 2,317 bladder cancer specimens (Ta to T3) revealed that while low cyclin E expression was generally associated with poor overall survival, it had virtually no prognostic value when analyzed independently of the tumor stage (Richter et al. 2000). However, Kamai et al. (2001) have reported that low cyclin E expression in a cohort of 145 consecutive bladder cancer patients was an independent predictor of overall survival. Thus, the prognostic value of cyclin E expression remains to be confirmed with further investigations.

### Cell adhesion molecules

High levels of matrix metalloproteinase–2 (MMP-2), tissue inhibitors of matrix metalloproteinase–2 (TIMP-2), and membrane-type MMP-1 expression have been strongly associated with decreased survival (Kanayama et al. 1998). However, it remains unknown whether the balance between the activity of MMPs and that of their inhibitors in serum or tissues can predict progression in patients with bladder cancer.

Reduced E-cadherin expression appears to be generally associated with increased muscle invasion and distant metastasis as well as with higher pathologic grades and stages (Bringuier et al. 1993; Otto et al. 1994). High E-cadherin expression has also been associated with improved overall survival and recurrence-free survival (Bringuier et al. 1993; Lipponen and Eskelinen, 1995; Ross et al. 1995; Syrigos et al. 1998). Moreover, Byrne et al. (2001) have reported that altered IHC-detected E-cadherin expression is significantly associated with disease progression and cancer-specific survival, and that E-cadherin and stage are independent predictors of disease progression. Thus, E-cadherin expression may be a promising prognostic variable. Another potential prognostic marker may be CD44, which is a widely expressed cell surface adhesion molecule that is involved in cell-cell and cell-matrix interactions. Two studies have shown that CD44 overexpression is closely associated with bladder cancer progression (Matsumura et al. 1995; Miyake et al. 2002).

In summary, the observations described above reveal a number of significant correlations between various molecular markers and tumor progression. In particular, p53, Ki-67, Rb, EGFR, E-cadherin and several cyclins appear to be of prognostic value with regard to bladder cancer metastasis, recurrence, and overall and cancer-specific survival. However, none of these markers are currently being utilized in clinical practice and their usefulness as independent prognosticators remains to be confirmed by large prospective comparative studies. More reliable and accurate methods to detect these markers should also be developed to improve their utility in the clinic.

## Epigenetic Markers

While genetics refers to the study of information inherited on the basis of gene sequence, epigenetics is the study of reversible changes that can be inherited in gene function or other cell phenotypes that occur without any change in DNA sequence. One example of such epigenetic changes is DNA methylation, which can induce different gene expression patterns in a tissue-specific and developmental-stage-specific manner. DNA methylation occurs throughout the genome and involves the addition of a methyl group to the cytosine ring of the CpG dinucleotide. The methylation process is catalyzed by an enzyme called DNA methyltransferases and it results in formation of methyl cytosines. DNA methylation within a promoter region is associated with silencing of the affiliated gene. The methylation pattern is established during development and is normally maintained throughout the life of an individual. Thus, DNA methylation is a key regulator of gene transcription and genomic stability and inappropriately altered DNA methylation pattern is a frequently detected epigenetic change in human cancers (Table 2).

In carcinogenesis, the mechanisms that generally regulate normal DNA methylation pattern are impaired and many cancers consequently show global hypomethylation accompanied by regional hypermethylation found in some promoter sequences. Thus, the detection of aberrant DNA promoter hypermethylation in cancers may be of considerable prognostic value (Dulaimi et al. 2004), particularly when the aberrant hypermethylation silences tumor suppressor genes (Herman and Baylin, 2003). For example, hypermethylation of the *Rb* tumor suppressor gene has been strongly connected with tumorigenesis because only about 9% of retinoblastomas nomally exhibit hypermethylation of their 5′ regions (Ohtani-Fujita et al. 1997). Since the promoters of many tumor suppressor genes contain CpG islands and show evidence of methylation-specific silencing, abnormal methylation of CpG islands may act as a key tumorigenic “hit” (Baylin and Herman, 2000; Jones and Laird 1999). Consequently, analyses of altered DNA methylation in cancers often focus on the CpG islands in the promoters of tumor suppressor genes.

As promoter hypermethylation occurs frequently in bladder cancer (Catto et al. 2005), several authors have investigated its presence in exfoliated urinary cells or tumor tissues (Kim et al. 2005; Maruyama et al. 2001; Chan et al. 2002; Dulaimi et al. 2004; Friedrich et al. 2004; Yates et al. 2006). Kim et al. (2005) demonstrated that methylation of *RUNX3* promoter sequence confers a 100-fold increased risk of developing bladder cancer (OR, 107.55). *RUNX3* methylation also appears to be positively associated with cancer stage (OR, 2.95), recurrence (OR, 3.70), and progression (OR, 5.63), which suggests that RUNX3 not only inhibits cancer initiation but also suppresses the aggressiveness of primary bladder cancers. Thus, the methylation status of *RUNX3* may be a better diagnostic marker for bladder cancer than previously described markers.

Catto et al. (2005) have analyzed the hypermethylation at 11 CpG islands in a large cohort of urothelial carcinomas. Compared with unmethylated tumors, methylation at these sites was significantly associated with advanced stage, high tumor progression rates, and increased mortality rates. These findings strongly suggest that patterns of promoter hypermethylation may actually be a cause of bladder cancer, at least in part. Thus, analysis of the methylation status may be useful as a diagnostic and prognostic marker for bladder cancer. Moreover, reversing aberrant methylation may be an effective way to treat bladder cancer.

Dulaimi et al. (2004) compared the matched sediment DNAs from urine specimens obtained before surgery with normal and benign control DNAs for the methylation status of three tumor suppressor genes, namely, *APC*, *RASSF1A*, and *p14^ARF^*. Hypermethylation of at least one of these genes was found in the matched urine DNA from 39 of 45 patients (87% sensitivity; 100% specificity), including 16 cases that had negative cytology. Indeed, hypermethylation (91%) in the urine DNA was more commonly detected than positive cytology (50%). Similarly, another group has also investigated the DNA methylation status of various apoptosis-associated genes (*DAPK*, *BCL2*, *TERT*, *EDNRB*, *RASSF1A*, and *TNFRSF25*) in urine sediments (Friedrich et al. 2004) and found that hypermethylation of the *DAPK*, *BCL2*, and *TERT* genes was highly sensitive (78%) and specific (100%) in detecting bladder cancer. Thus, the detection of DNA hypermethylation in voided urine is promising in terms of the early detection and surveillance of bladder cancer.

There are, however, a number of criticisms of the clinical and prognostic relevance of assays detecting promoter hypermethylation in bladder cancer. Firstly, some markers, such as *RASSF1*, can be commonly observed in primary tumors including lung, breast, pancreas, kidney, liver, cervix, nasopharyngeal, prostate, thyroid and other cancers. Moreover, *RASSF1A* methylation was frequently detected in body fluids including blood, urine, nipple aspirates, sputum and bronchial alveolar lavages. Inactivation of RASSF1A was associated with an advanced tumor stage (e.g. bladder, brain, prostate, gastric tumors) and poor prognosis (e.g. lung, sarcoma and breast cancer). Detection of aberrant *RASSF1A* methylation may serve as a diagnostic and prognostic marker (Damman et al. 2005; Maruyama et al. 2005). Even though a specific methylation marker in bladder cancer might be promising in terms of its prognostic value, the most powerful detection tools established up to date, such as cystoscopy in bladder cancer and PSA in prostate cancer should be firstly considered and methylation markers must be used to support the function of primary detection methods. In the near future, it may be possible that technologies will become available to characterize different methylation patterns related to various cancer types. Secondly, several studies of promoter methylation produced largely contradictory results, which weakened the argument for the inspection of promoter methylation patterns as a prognostic method. Eventually, new methodologies might explain these discrepancies, but these dissimilarities emphasize the need for standardized methodological protocols if molecular diagnostic tools are to be a useful component of routine clinical practice. Additionally, discrepancies between methylation profiles and gene expression have been reported, thus raising the issue of whether DNA methylation is the cause of gene down regulation. Earlier, we reported strong evidence suggesting that *RUNX3* is a bladder cancer tumor suppressor gene and that it is frequently inactivated by hypermethylation of the promoter region (Kim et al. 2005). Therefore, promoter hypermethylation, including that of *RUNX3*, could induce not only gene down regulation, but also tumorigenesis.

## Gene Expression Profiles as Promising Bladder Cancer Markers

New high-throughput microarray technologies have made it possible to gain a comprehensive insight into the molecular basis of many human diseases (Bubendorf, 2001). In particular, searching for the genes that are differentially expressed in human cancers has been greatly facilitated by DNA microarray technology. With this technology, the RNA expression levels of hundreds or even thousands of genes in a tumor can be surveyed simultaneously. The tissue micro-array technology has also been developed to enable a large-scale molecular analysis of hundreds of tumor samples. Oligonucleotide arrays, such as those manufactured by Affymetrix (www.affymetrix.com) or Illumina (www.illumina.com), represent some standardized approaches that are being widely accepted. These microarrays consist of grids bearing thousands of 20–25 or 50 bp oligonucleotides that have been selected from known sequences by design algorithms that choose probes that hybridize to their complements with high affinity and specificity (Lipshutz et al. 1999; Lockhart et al. 1996). Each fluorescently labeled sample of mixed nucleic acids is hybridized to the array individually. This results in the quantitation of absolute gene expression levels. In contrast, cDNA microarrays require fluorescent labeling of nucleic acids isolated from a test sample (e.g. bladder cancer tissue) and differently labeled nucleic acids from a reference sample (e.g. normal bladder tissue) (Duggan et al. 1999; Schena et al. 1995). The results thus generate ratios of gene expression of the test sample relative to that of the reference sample. cDNA arrays are commercially available or may be fabricated specifically to assay the experimental system in question (Cheung et al. 1999).

Recently, Illumina has generated two kinds of BeadChips to analyze human gene expression. The HumanRef-8 Expression BeadChip, which has >24,000 probes, generates the whole-genome expression profiles of eight samples on a single BeadChip. The HumanRef-8 BeadChip array represents genome-wide transcriptional coverage of well-characterized National Center for Biotechnology Information (NCBI) Reference Sequence (RefSeq; Release 4) genes. These RefSeq genes are validated, annotated, and curated on an ongoing basis by NCBI staff and collaborators. The Human-6 BeadChip generates the whole-genome expression profiles of six samples on a single BeadChip. Each Human-6 BeadChip microarray contains >47,000 probes and targets >46,000 human transcripts and represents genome-wide transcriptional coverage of well-characterized genes, gene candidates, and splice variants. The per-sample probe content is designed and assembled hierarchically. One stripe in each array pair is based on the same RefSeq genes found in the HumanRef-8 BeadChip, while the second stripe includes probes for genes with unique locations in the human genome, such as expressed sequence tags (ESTs) or those that appear in multiple independent databases. The probes are specifically designed to avoid querying pseudogenes and SNP sites. These microarray technologies are and will be of considerable value in the urological field. In particular, microarray gene expression analysis is likely to greatly facilitate the identification of molecular prognostic markers that correlate with particular bladder cancer outcomes.

Bladder cancer frequently occurs as multifocal disease involving several simultaneous tumors scattered over the bladder. Modlich et al. (2004) have reported that multifocal superficial bladder cancers always cluster together, which suggests they share a common genetic background. The clonality of multifocal bladder cancer has also been reported by several other studies, which are based on comprehensive LOH analyses (Hartmann et al. 2000; Sidransky et al. 1992). Tissue RNA expression microarrays of the multifocal tumors would be useful in confirming the genetic homogeneity of multifocal tumor cells.

Bladder cancers also present as invasive cancers, which are histologically complex tissues that consist of a variety of cell types other than carcinoma cells. As with other methods, tissue RNA expression microarrays determine the expression levels irrespective of cell types. Despite this, tissue RNA expression microarray analyses of invasive bladder cancers may also offer some important clinical information. This is due to the fact that tumor development and progression (e.g. growth, angiogenesis, tumor cell detachment, and invasion) are dependent on interactions between tumor cells and the host microenvironment (stroma). Hence, the expression of factors involved in tumor-cell stroma interactions may reflect important biological properties that are independent of cellular origin (Primdahl et al. 2002; Keleg et al. 2003; Hsu et al. 2002; Liotta and Kohn, 2001; Seripa et al. 2001). Indeed, while microdissection would allow the examination of highly pure tumor cell populations of a given specimen, it might not reflect the tumor itself *in vivo*. Thus, identification of a molecular classifier gene set, which is to certain extent independent of tumor tissue heterogeneity, is required.

With regard to the microarray studies that have been performed in the bladder cancer field to date, Sanchez-Carbayo et al. (2003) have used cDNA microarrays to facilitate the hierarchical clustering of superficial and invasive bladder cancers. This technology allowed them to separate carcinoma *in situ* from papillary superficial lesions, and to identify subgroups within superficial and invasive cancers that differ in overall survival. The most extensive expression profiling study of bladder cancers reported to date is by Dyrskjot and coworkers (Dyrskjot, 2003; Dyrskjot et al. 2003), who identified clinically relevant subclasses of bladder cancer on the basis of their expression microarray profiles. Cluster analysis identified three major stages, namely, Ta, T1 and T2-4, with the Ta tumors being further classified into subgroups. In particular, a 32-gene molecular classifier set of genes could be used to classify benign and muscle invasive cancers with a close correlation to pathological staging. This analysis also provided new predictive information on disease progression in Ta tumors as compared with conventional staging. Furthermore, the gene expression profiles that characterized each stage and subtype revealed their biological properties, thereby identifying new potential targets for therapy. Dyrskjot et al. (2004) then extended their work by examining the gene expression patterns of muscle invasive carcinomas and superficial transitional cell carcinomas with and without surrounding carcinoma *in situ*. The transitional cell carcinomas with surrounding carcinoma *in situ* and invasive carcinomas showed similar expression levels of a few gene clusters. As a result, a 16-gene molecular classifier that represents the bladder carcinoma *in situ* gene expression signature was constructed. This classifier was suggested to be useful in the follow-up of bladder cancer patients.

Modlich et al. (2004) subjected tumor specimens of a cohort of uniformly treated patients with a well-defined clinical outcome to hierarchical cluster analysis. Superficial and invasive tumors were found to display distinct gene expression profiles. Moreover, distinct prognostic groups of invasive bladder cancer could be identified. When different subsets of gene expression data, including a set of 41 genes, were analyzed by using two different array platforms, the gene and tumor clustering patterns were remarkably stable. Superficial tumors were also found to be of clonal origin, and different areas of invasive tumors showed highly similar gene expression patterns. Moreover, the bladder mucosa of patients with locally advanced disease was shown to express an invasive type of pattern. These data thus provide additional insights into the molecular pathogenesis of bladder cancer and help detect novel prognostic markers for superficial, invasive, and metastasizing disease.

Blaveri et al. (2005) have characterized the global gene expression patterns in 80 bladder cancers, 9 bladder cancer cell lines, and 3 normal bladder samples by cDNA microarrays containing 10,368 human gene elements. Unsupervised hierarchical clustering successfully separated the samples into two subgroups that contained superficial (pTa and pT1) or muscle invasive (pT2–pT4) tumors. Supervised classification based on a limited subset of genes had a 90.5% success rate in separating superficial from muscle invasive tumors. Tumors could also be classified into transitional versus squamous subtypes (89% success rate) and a good versus bad prognosis (78% success rate). It was concluded that the genes driving the separation between tumor subsets may be important biomarkers for bladder cancer development and progression. Furthermore, these biomarkers could be candidates for therapeutic targeting.

The full utility of microarray analyses in bladder cancer research, diagnosis, prognosis, and treatment remains to be determined by additional clinical trials. One particularly critical issue that should be addressed by microarray analyses is the identification of superficial disease subtypes and the patients who are more likely to develop positive lymph nodes or distant metastases. It is likely that in the near future, gene profiling will be an effective way of predicting the response to specific therapeutic regimes, as it will determine the molecular signatures of the tumors with respect to their chemosensitivity or resistance to anticancer drugs. Moreover, the discovery of prognostic markers in cancer progression, as well as the identification of molecule-susceptible targets, will lead to the development of novel alternative therapies. Thus, the classical concept of the tumor marker is currently being expanded from an individual biological determinant to gene clusters that can act as predictive classifiers.

## Figures and Tables

**Table 1:** Potential genetic markers in bladder cancer.

Markers	Locus	Methods	Potential prognostic value	Reference
**Proto-oncogenes/Oncogenes**				
*EGFR*	7p12	IHC	High grade/high stage	(Neal et al. 1985; Messing1990; Nguyen et al. 1994; Lipponen and Eskelinen, 1994)
*HER-2/neu* (*c-erb-B2*)	17q21.1	IHC	High grade/high stage/poor survival/metastasis	(Lipponen et al. 1991; (Sato et al. 1992))
*FGFR3*	4p16.3	PCR	Low grade/low stage/prognosis (recurrence, progression, survival)	(Rhijn et al. 2001; van Rhijn et al. 2003)
*c-myc*	8q24	IHC	No association with recurrence, progression, or survival	(Kotake et al. 1990; Lipponen, 1995; Schmitz-Drager et al. 1997)
**Tumor Suppressor Genes**				
*p53*	17p13.1	IHC	High stage/prognosis (recurrence, progression, survival)/Resistance to chemotherapy	(Schmitz-Drager et al. 2000; Sarkis et al. 1995; Sengelov et al. 1997)
*Rb*	13q14.2	IHC	High stage/prognosis (recurrence, progression, survival)	(Cordon-Cardo et al. 1992; Logothetis et al. 1992; Cote et al. 1998)
**Cell Cycle Regulators**				
p21	6p21.2	IHC	High stage/prognosis (recurrence, survival)	(Stein et al. 1998)
p27	12p13.1	IHC	High grade/survival	(Korkolopoulou et al. 2000)
Ki-67	10q25	IHC	Progression/recurrence	(Gerdes et al. 1984)
cyclin D1	11q13	IHC	Low grade/low stage/recurrence	(Wagner et al. 1999; Liukkonen et al. 2000)
cyclin E	19q12	IHC	Low stage/survival	(Richter et al. 2000; Kamai et al. 2001)
**Cell Adhesion Molecules**				
MMP-2	16q13	PCR	High stage/survival	(Kanayama et al. 1998)
E-cadherin	16q22.1	IHC	High grade/high stage/progression/survival	(Bringuier et al. 1993; Otto et al. 1994; Lipponen and Eskelinen, 1995; Ross et al. 1995; Syrigos et al. 1998) Byrne et al. 2001)
CD44	11p13	PCR	High stage/survival	(Matsumura et al. 1995; Miyake et al. 2002)

IHC: Immunohistochemistry

PCR: polymerase chain reaction

**Table 2:** Potential epigenetic markers in bladder cancer.

Study	No.	Method	Chromosomal locus	Methylation markers	Methylated rate (%)		Potential prognostic value
					Tissue	Urine	
Catto et al. 2005	280	MSP	3p21 9q34.1	*RASSF1A* *DAPK*	59 6		High grade/high stage/progression/survival
Chan et al. 2002	98	MSP	9q34.1 3p24.1 16q22.1 9p21	*DAPK* *RAR*β *E-cadherin* *p*^16^	58.2 87.8 63.3 26.5	45.5 68.2 59.1 13.6	No association with grade and stage Detection of gene methylation in urine is more sensitive than conventional urine cytology
Dulaimi et al. 2004	45	MSP	5q21-q22 3p21 9p21	*APC* *RASSF1A* *p14*^ARF^		69 51 35	No association with grade and stage Detection of gene methylation in urine is more sensitive than conventional urine cytology
Friedrich et al. 2004	125	QMSP	9q34.1 18q21.3 5p15.33	*DAPK* *BCL2* *TERT*	NA 52 25.2	21.6 64.9 51.4	High grade/high stage Promising tools for noninvasive detection of bladder cancer
Kim et al. 2005	124	MSP	1p36	*RUNX3*	73		Tumor development/recurrence/progression
Maruyama et al. 2001	98	MSP 3p21	16q22.1 *RASSF1A* 5q21-q22 16q24.2-q24.3 3p14.2	*CDH1* 35 *APC* *CDH13* *FHIT*	36 35 29 16		Poor prognosis (grade, growth pattern, muscle invasion, tumor stage, and ploidy pattern)/Survival
Yates et al. 2006	35	QMSP	3p21 16q22.1 5q21-q22	*RASSF1A* *E-cadherin* *APC*		51 31 40	Detection of gene methylation in urine are associated with age and malignancy

MSP: Methylation-specific PCR

QMSP: Quantitative methylation-specific

PCR NA: Not available
